# 8-Aminoguanine protects against paclitaxel-induced neural degeneration and mechanical allodynia

**DOI:** 10.1172/jci.insight.202639

**Published:** 2026-04-08

**Authors:** Lori A. Birder, Amanda Wolf-Johnston, Jonathan Franks, Mara L.G. Sullivan, Simon C. Watkins, Anthony J. Kanai, Vladimir B. Ritov, Edwin K. Jackson

**Affiliations:** 1 Renal-Electrolyte Division, Department of Medicine,; 2 Department of Pharmacology and Chemical Biology, and; 3 Department of Cell Biology, University of Pittsburgh School of Medicine, Pittsburgh, Pennsylvania, USA.

**Keywords:** Cell biology, Neuroscience, Oncology, Breast cancer, Mitochondria, Neurodegeneration

## Abstract

Current treatment protocols for most types of cancers require chemotherapeutic agents that are associated with significant side effects, including chemotherapy-induced peripheral neuropathy (CIPN). Currently, there are no effective CIPN prevention strategies, and current treatment approaches remain limited. The enzyme purine nucleoside phosphorylase (PNPase) actively modulates both oxidative injury and cellular damage. Here, we tested the hypothesis that the signs and symptoms of CIPN are due to a chemotherapy-induced dysregulation of the purine metabolome. We assessed the effect of PNPase inhibition on paclitaxel-induced (PAC-induced) CIPN. Female adult Sprague-Dawley rats were treated with PAC and randomized to oral treatment with either the PNPase inhibitor 8-aminoguanine (8-AG) or its vehicle. Some rats were injected with shRNA against PNPase prior to PAC injections. PAC-treated rats exhibited multiple abnormalities: mechanical allodynia and changes in damaging purines, intraepidermal nerve fiber (IENF) density, and signaling cascades involved in mitochondrial disruption and axonal damage. Inhibition of PNPase improved behavioral function (mechanical allodynia), rescued the loss/damage of IENF, and normalized markers for mitochondrial dysfunction and nerve damage. These findings support the hypothesis that inhibition of PNPase prevented (and potentially reversed) CIPN through several mechanisms that included a reduction in neuronal damage and development of mechanical allodynia.

## Abstract 2

Our findings show that the signs and symptoms of CIPN are due to a dysregulation of the purine metabolome because of an increase in PNPase activity; decreasing PNPase activity prevents (and potentially reverses) CIPN .

## Introduction

Chemotherapy-induced peripheral neuropathy (CIPN) is the most common and dose limiting side effect of antineoplastic treatments, reducing both the therapeutic potential of these life-saving drugs and patients’ quality of life. There are currently no effective preventative measures nor any effective treatment options (1). Current drugs and therapies that target the signs and symptoms of CIPN are often ineffective and frequently linked to serious adverse effects, leading to poor patient adherence and physician and patient dissatisfaction (2).

The epidermis is innervated by sensory fibers that cross the dermal-epidermal border (intraepidermal nerve fibers [IENFs]), and the loss of these fibers has been associated with neuropathic symptoms, including mechanical hypersensitivity. While a number of mechanisms have been suggested to account for the chemotherapy-induced damage to sensory nerve endings (2–4), a prevailing hypothesis is that the nerve fiber loss is due to mitochondrial dysfunction, which causes the generation of ROS, leading to oxidative stress. Peripheral nerves are highly susceptible to free radical damage owing in part to weak neuronal antioxidant defense mechanisms (5). Increased production of ROS occurs in many chronic pain conditions, and consequently, ROS production and associated oxidative stress remain important therapeutic targets (6, 7).

Although the mechanisms of ROS-induced nerve damage are not completely understood, emerging evidence suggests that alterations in the activity of the enzyme purine nucleoside phosphorylase (PNPase, an enzyme at the crossroads of purine biochemistry) contributes to oxidative injury and cellular damage (8, 9). PNPase belongs to the family of glycosyltransferases, is expressed in both bacteria and mammals, and is one of the key enzymes involved in the purine salvage pathway (10, 11). PNPase transforms protective purines (e.g., inosine and guanosine) to “tissue damaging” purines (i.e., hypoxanthine [HX] and xanthine), which may exhibit harmful effects due to production of free radicals (specifically, superoxide anion [O^–^_2_] and hydrogen peroxide [H_2_O_2_]) when HX is metabolized by xanthine oxidase (XO) to xanthine and hence to uric acid (12–14). ROS formed via the HX/XO mechanism likely would damage mitochondria (15–18). Since damaged mitochondria per se are a major cellular source of ROS, this mechanism would represent a feed-forward process that amplifies HX/XO-induced ROS production. Importantly, superoxide and peroxynitrite and their generation are key mediators in the development of peripheral and central sensitization of various pain etiologies, including CIPN (19). Thus, dysregulation of PNPase may shift a protective purine metabolome to a harmful one, potentially contributing to IENF loss and the onset of CIPN. Paclitaxel (PAC) is a widely used chemotherapeutic agent that often induces CIPN. To test our hypothesis that PNPase contributes to CIPN, here we tested whether inhibition of PNPase attenuates PAC-induced loss of IENFs and development of mechanical allodynia.

## Results

### HX, xanthine, and isoprostanes.

**Figure 1:**
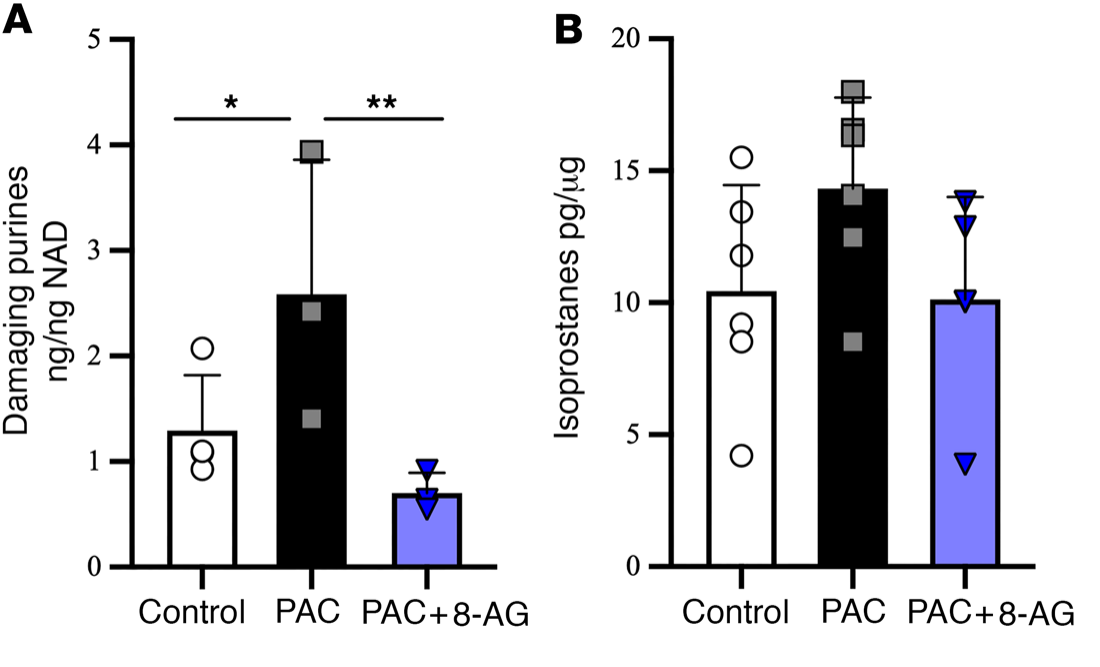
8-Aminoguanine attenuates PAC-related changes in proinflammatory purines and biomarkers for oxidative stress. Levels of neurodamaging purines (**A**, hypoxanthine and xanthine, *n* = 3–4 per group) were higher in sciatic nerves of rats treated with PAC yet were normalized in sciatic nerves of rats treated with PAC + 8-aminoguanine (8-AG). These purines were measured by UPLC-MS/MS in ng and normalized by tissue dry weight (mg) after lyophilization. Isoprostanes (**B**, *n* = 5–6 per group) were elevated in sciatic nerves of rats treated with PAC and normalized with 8-AG treatment. Isoprostanes were measured by ELISA as pg normalized to mg protein. Data are presented as mean ± SD. Ordinary 1-way ANOVA (**A**, *P* = 0.2712; **B**, *P* = 0.0081) followed by a Fisher’s least significant difference test between groups, *P < 0.05, **P < 0.01.

In PAC-treated rats, sciatic tissue levels of the neurotoxic purines HX and xanthine (Figure 1A) were significantly increased as compared with those of untreated controls. However, both were restored to control levels following oral 8-AG given 72 hours following PAC treatment (Figure 1). We also observed a trend toward increased levels of isoprostanes, used as a biomarker for increased ROS as an indicator of oxidative stress, which was reduced with 8-AG (Figure 1B).

### Mechanical allodynia.

**Figure 2:**
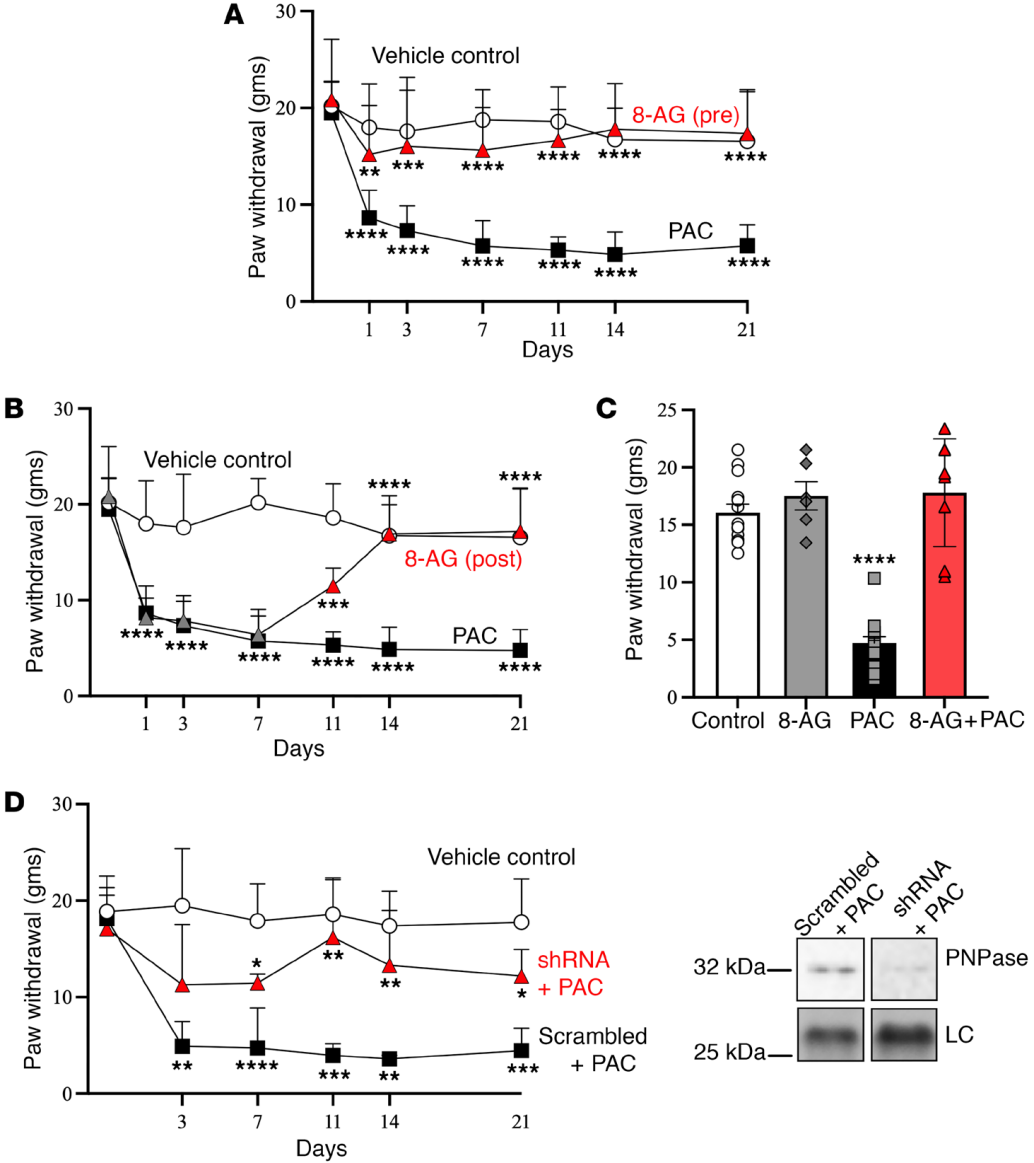
8-Aminoguanine attenuates PAC-related changes in mechanical sensitivity. PAC increased sciatic nerve responses to tactile mechanical stimuli, and this was prevented by 8-Aminoguanine (8-AG) (**A**, *n* = 6–21 per group) or restored to responses similar to that of untreated control rats by 8-AG given 72 hours following PAC treatment (**B**, n= 8-14 per group). 8-AG’s effect on mechanosensitivity was restricted to PAC-treated animals. We found a similar response in sciatic nerve responses to tactile mechanical sensitivity between vehicle controls and rats treated with 8-AG without PAC (**C**, *n* = 6 to 14). The 8-AG effect was restricted to PAC-treated rats; control rats were not affected by 8-AG. Two-way ANOVA (main effects and interactions, *P* < 0.0001) followed by Fisher’s least significant difference test show that PAC/8-AG group was significantly different from the other 3 groups but that the other 3 groups are not different from each other. PNPase knockdown attenuates PAC-related changes in nerve functions. PAC increased sciatic nerve responses to tactile stimuli relative to controls. This mechanical hypersensitivity was prevented (**D**, *n* = 4 per group) by injection of a lentiviral-based shRNA to knockdown the expression of PNPase in the sciatic nerve. Injections of sham viral particles (i.e., the shRNA cassette contained a scrambled sequence) had no effect on PAC changes in nerve functions. Representative immunoblots are shown; data are calculated as relative intensity normalized to total protein loading control (LC). All samples were run on the same blot, but representative samples were not contiguous. Data are presented as mean ± SD. Ordinary 1-way (**A**, *P* < 0.0001; **B**, *P* < 0.0001; **C**, *P* < 0.0001) ANOVA (followed by a Fisher’s least significant difference test between groups, *P < 0.05, ** *P* < 0.01, ***P < 0.001 ****P < 0.0001.

PAC treatment was associated with the emergence of mechanical allodynia, as manifested by a decrease in the mechanical withdrawal threshold (Figure 2A) (3). Consistent with findings from previously published reports (3, 33), PAC-treated rats exhibited an increase in tactile sensitivity that affected the hind paw from the first day until sacrifice (day 21). Importantly, PAC-induced mechanical allodynia was blocked by cotreatment with 8-aminoguanine (8-AG), started as pretreatment (14 days prior to start of PAC). Remarkably, 8-AG was also able to reverse established mechanical allodynia, as demonstrated in rats given 8-AG 72 hours after the last dose of PAC (Figure 2B). Notably, administration of 8-AG to control rats (i.e., rats not treated with PAC) had no effect on mechanical hypersensitivity (Figure 2C), indicating that the 8-AG effect was restricted to PAC-treated rats. As there was no difference between 8-AG alone and vehicle treatment, we utilized vehicle treatment in all subsequent experiments.

To further increase robustness of our inferences, we also tested the role of PNPase in CIPN mechanical allodynia using molecular approaches to knockdown PNPase in the sciatic nerve. Injecting into the sciatic nerve can deliver therapeutic agents directly to the primary site of injury, allowing for targeted investigation of potential treatments for CIPN. Here, a separate group of rats was pretreated with viral particles injected bilaterally into the sciatic nerve carrying either shRNA directed against PNPase mRNA or viral particles in which the shRNA cassette was replaced with a scrambled sequence (sham-treated animals, i.e., controls). shRNA knocked down PNPase by 73% ± 17% (Figure 2D; representative immunoblots are shown). We observed that shRNA knockdown of PNPase significantly reduced mechanical allodynia (Figure 2D) compared with PAC-treated rats without shRNA treatment.

### Changes in IENF density.

**Figure 3:**
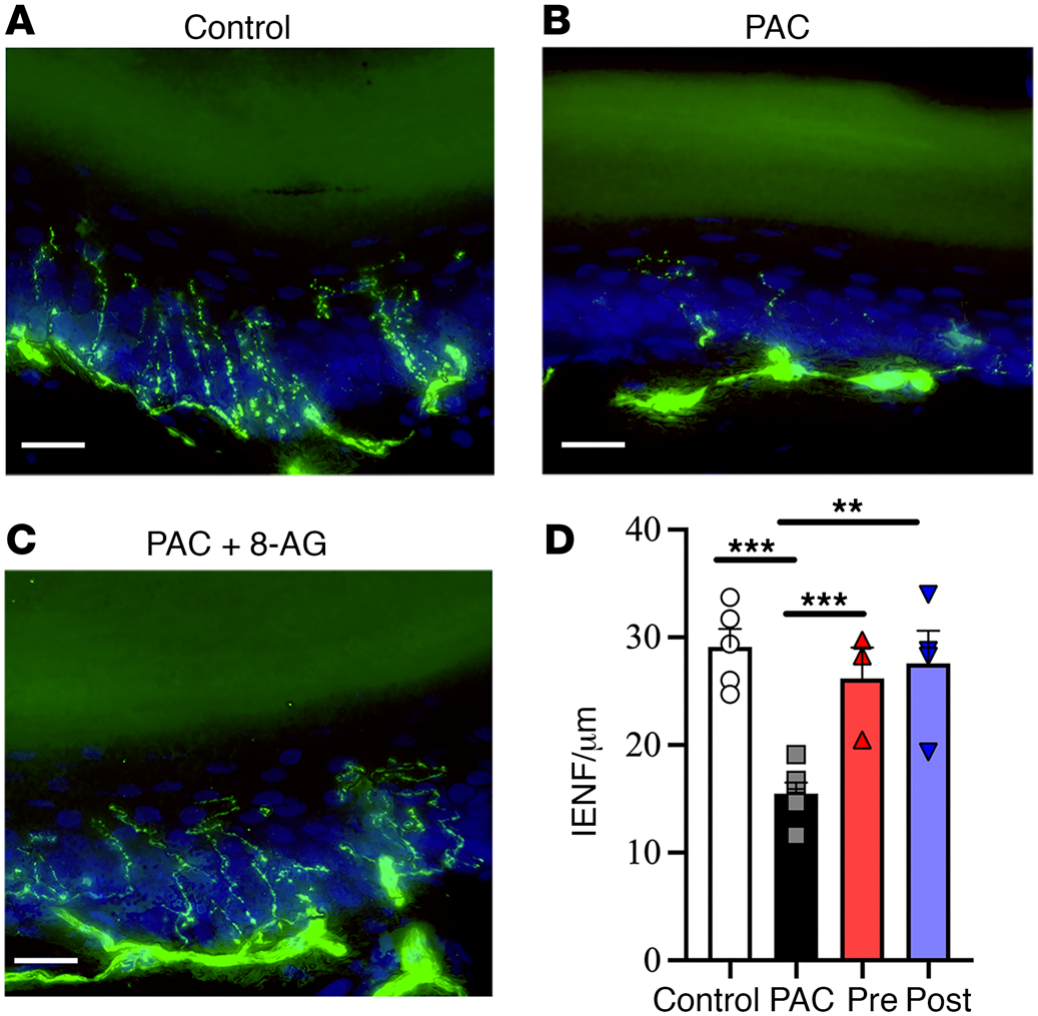
8-Aminoguanine attenuates PAC-related changes in intraepidermal nerve fibers. (**A**) Representative image from control rat glabrous skin whereby PGP-9.5–positive nerve fibers (green, intraepidermal nerve fibers [IENF]) can be seen crossing the basement membrane and extending into the epidermis. Rats treated with paclitaxel (PAC, *n* = 6 per group) exhibited fewer IENF (**B**) compared with controls (**A**, *n* = 5 per group) and rats treated with PAC + 8-aminoguanine (8-AG) (**C**). (**D**) Values were normalized to the longitudinal length of epidermis (μm) in each field. There was no significant difference in between 8-AG administered prior to PAC (Pre, *n* = 3 per group) or 8-AG given 72 hours following PAC (Post, *n* = 4 per group) treatment. For IENF quantification, a minimum of 5 plantar skin sections per animal with 3 sites were examined. Scale bars: 20 μm. Nuclei (blue) were stained with DAPI. Data are presented as mean ± SD. Ordinary 1-way ANOVA (*P* = 0.0004) followed by Fisher’s least significant difference test between groups, **P < 0.01, ***P < 0.001.

**Figure 4:**
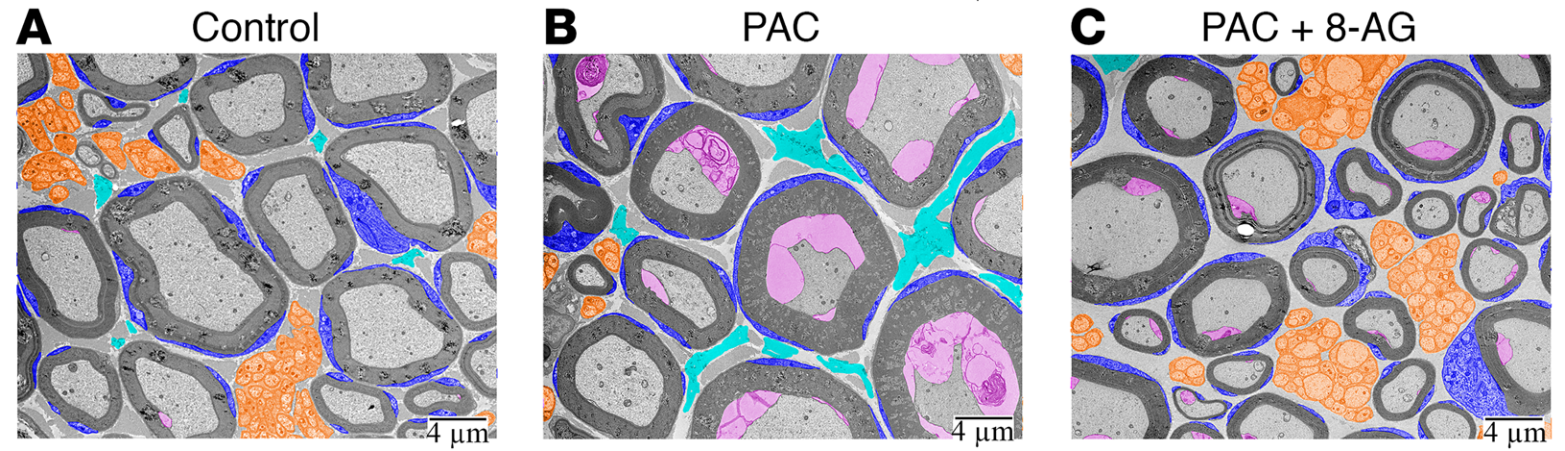
8-Aminoguanine attenuates PAC-related changes in small-diameter fibers in rat sciatic nerves. Large area scanning electron microscopy approach was used to scan the entire thin section of the sciatic nerve. (**A**) Representative image whereby (**B**) PAC treatment results in a notable decrease in small-diameter fibers (orange), along with increased space between myelinated nerves (cyan), and a delamination of the inner mesaxon inside the myelinated nerve (magenta) relative to control (**A**). (**C**) Importantly, 8-aminoguanine (8-AG) treatment restores these deficits to that of a control state. *n* = 4 per group; Scale bars 4 μm.

IENF density was quantified by normalizing the number of epidermal nerve fibers in each mm of dermal length in a minimum of 3 images/section. Consistent with previous studies (3), we found that relative to untreated control rats (Figure 3A), IENF density was significantly reduced in PAC-treated rats (Figure 3B). This PAC-induced reduction in IENF density was blocked by 8-AG treatment (Figure 3, C and D). We also found that 8-AG administered 72 hours following the last dose of PAC restored IENF density to that of a control state (Figure 3D). We used large area scanning electron microscopy approaches to scan the entire thin section of the sciatic nerve. Here, we provide compelling evidence (Figure 4) of a notable decrease in small-diameter fibers in PAC-treated rat sciatic nerves relative to those of controls, with coadministration of 8-AG restoring these changes to a control state.

### Gap43 immunofluorescence.

**Figure 5:**
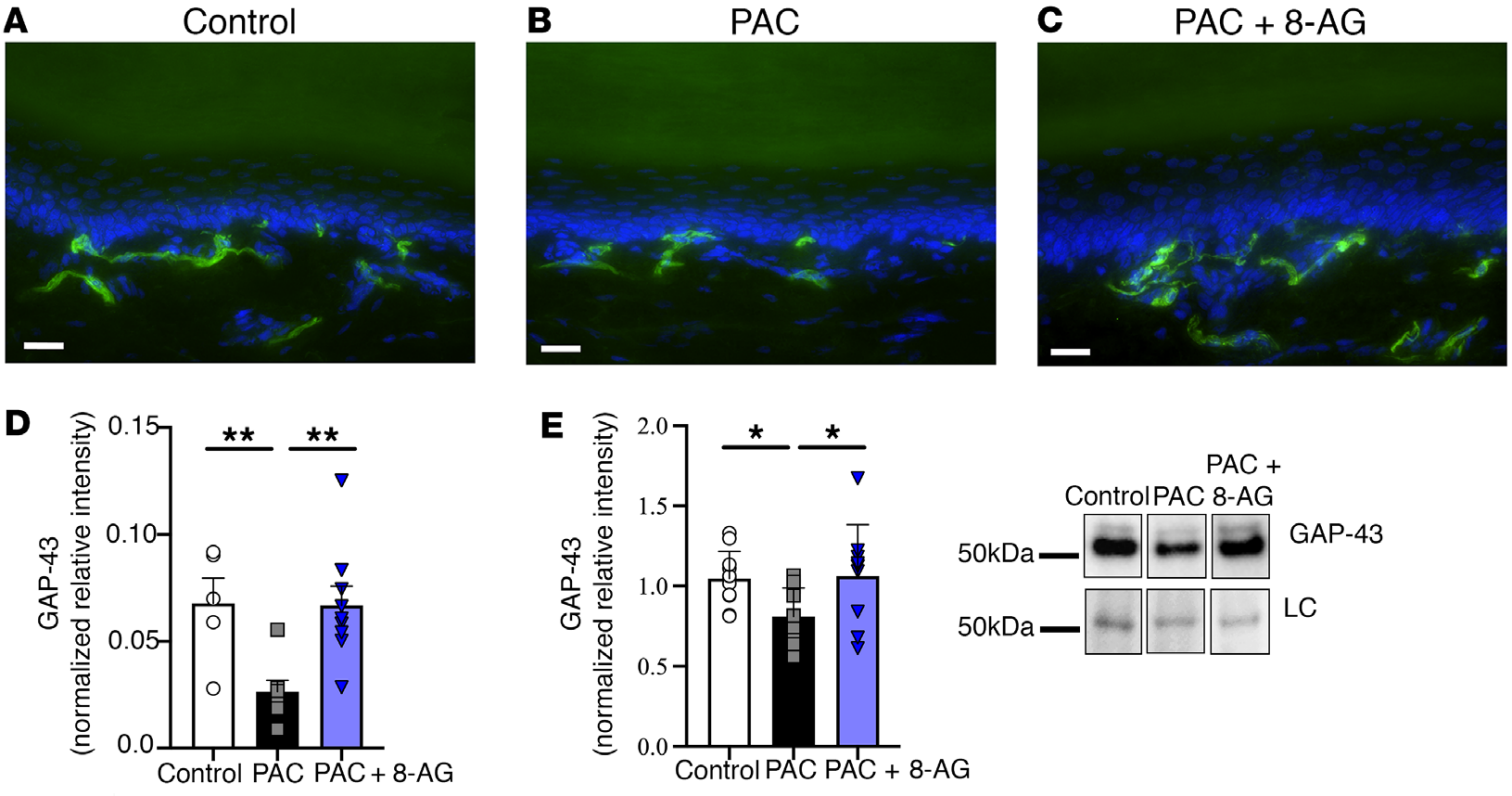
8-Aminoguanine facilitates nerve regeneration following PAC treatment. Representative image showing fluorescence labeling with GAP-43 (green) from control rat (**A**, *n* = 5) and after treatment with PAC (**B**, *n* = 7). Representative image (**C**, *n* = 9) from a PAC-rat that received 8-aminoguanine (8-AG) 72 hours after PAC treatment until sacrifice (day 21). Scale bars: 20 μm. Nuclei (blue) were stained with DAPI. (**D**) GAP-43 quantification, with a minimum of 5 plantar skin sections per animal with 3 sites for each section (*n* = 5–9). (**E**) Western immunoblotting shows significant alterations by PAC in the expression of GAP-43; treatment with 8-AG (72 hours following PAC) blocked the PAC-induced changes, such that PAC + 8-AG–treated sciatic nerves were similar to those of controls. A representative immunoblot of GAP-43 is shown; data are calculated as relative intensity normalized to total protein loading control (LC). All samples were run on the same blot, but representative samples were not contiguous. Data are presented as mean ± SD. Ordinary 1-way ANOVA (**D**, *P* = 0.0051; **E**, *P* = 0.0259) followed by a Fisher’s least significant difference test between groups, *P < 0.05, **P < 0.01.

Growth associated protein-43 (GAP-43) staining is a widely used method to visualize nerve regeneration and plasticity after nerve injury (20, 21). We found that GAP-43 immunofluorescence of intraepidermal innervation in the hind plantar paw skin was significantly decreased (day 21) following PAC treatment as compared with that of controls (Figure 5, A and B). However, administration of 8-AG 72 hours after the last dose of PAC increased GAP-43–positive IENFs (Figure 5C) compared with PAC rats at a similar time point. GAP-43 fluorescence was evaluated in a minimum of 3 images/section using ImageJ (NIH) and normalized to the mean relative intensity of the same area (Figure 5D). We also observed a significant decrease in GAP-43 expression in the sciatic nerve following PAC treatment as compared with that in controls (Figure 5E, representative Western blots). Administration of 8-AG 72 hours following the last dose of PAC increased GAP-43 expression compared with PAC rats at a similar time point.

### Proteins involved in neuronal plasticity.

**Figure 6:**
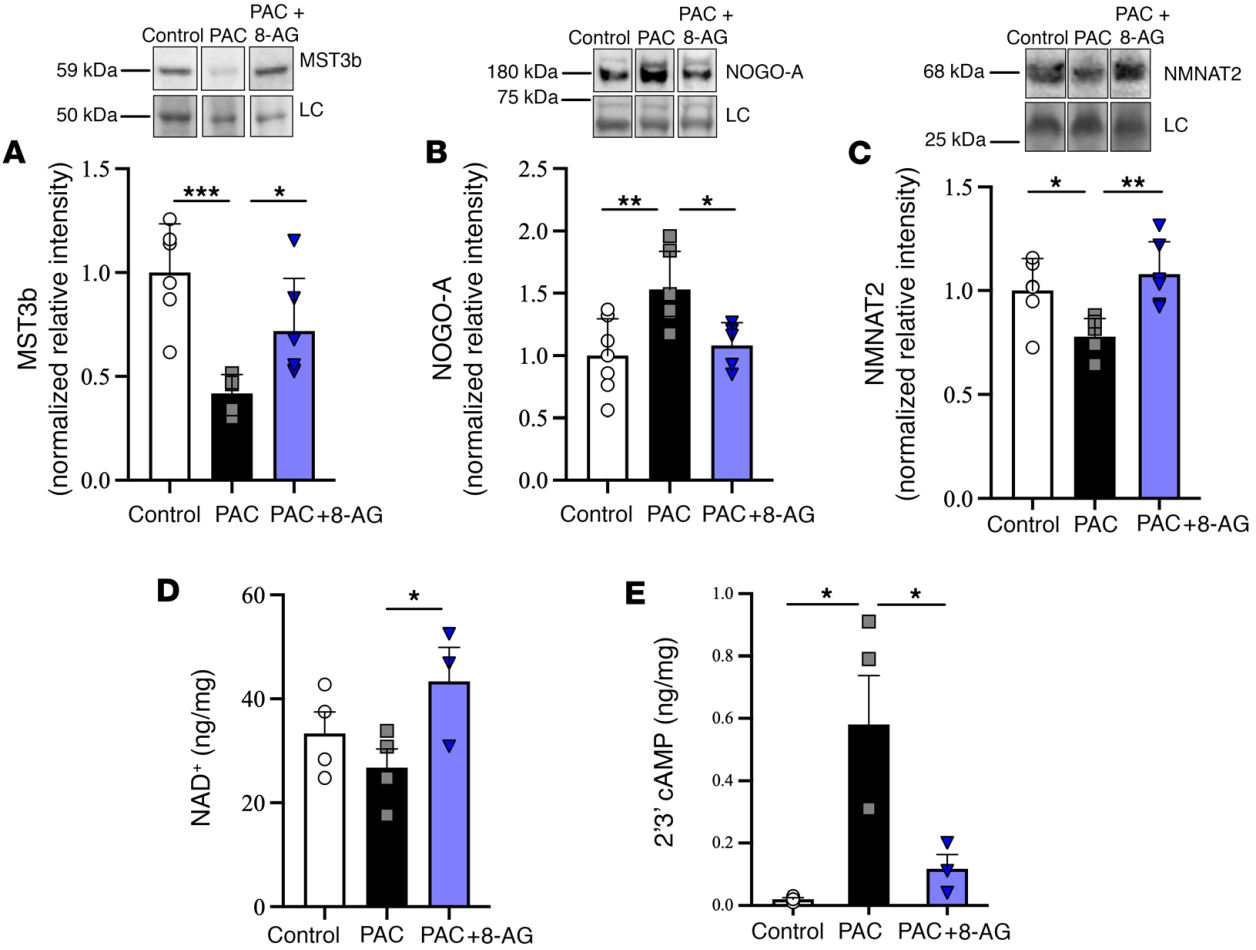
8-Aminoguanine restores PAC-associated changes in proteins associated with nerve growth and regeneration. (**A**) Western immunoblotting shows significant alterations by PAC in the expression of Mst3b (*n* = 5–6 per group), a biomarker for axon regeneration; (**B**) Nogo-A (*n* = 5–7 per group), a negative regulator of neurite growth and axonal regeneration; and (**C**) NMNAT2 (*n* = 6 per group), a protein necessary for maintaining healthy axons and a trend toward decrease in (**D**) NAD^+^ (*n* = 3–4 per group), a coenzyme needed for energy metabolism. (**E**) PAC also significantly increases the cyclic nucleotide 2’3’-cAMP (*n* = 5 per group), which enhances the opening of the mPTP. In all cases, treatment with 8-AG (72 hours following PAC) mitigated the PAC-induced changes, such that PAC + 8-aminoguanin3e (8-AG) sciatic nerves were similar to control, healthy sciatic nerves. Data are calculated as relative intensity normalized to total protein loading control (LC). Representative immunoblots are shown. All samples were run on the same blot, but representative samples were not contiguous. Data are presented as mean ± SD. Ordinary 1-way ANOVA (**A**, *P* = 0.018; **B**, *P* = 0.0084; **C**, *P* = 0.0050; **D**, *P* = 0.100; E, *P* = 0.020). followed by a Fisher’s least significant difference test between groups, *P < 0.05, **P < 0.01, ***P < 0.001.

Mammalian sterile 20-like kinase-3b (Mst3b/Stk24) has been shown to regulate the axon-promoting effects of trophic and other factors (22). Consistent with studies that show suppression of Mst3b expression blocks axonal outgrowth (22, 23), we found that, in sciatic nerves from PAC-treated rats, Mst3b expression was significantly decreased relative to that in controls (Figure 6A). However, 8-AG given 72 hours following the last dose of PAC restored Mst3b expression to that of a vehicle control (Figure 6A).

Neurite outgrowth inhibitor-A (Nogo-A) has been shown to be increased after nerve injury and inhibits axonal regeneration (24, 25). The protein expression of Nogo-A (detected by Western blot) was significantly elevated in the sciatic nerve relative to that in controls (Figure 6B). In sciatic nerves, 8-AG administered 72 hours following the last dose of PAC significantly decreased Nogo-A expression compared with that in the control group (Figure 6B).

In PAC-treated rats, levels of nicotinamide mononucleotide adenylyltransferase 2 (NMNAT2), a highly conserved nicotinamide adenine dinucleotide (NAD^+^) synthase that plays an important role in neuroprotection, were decreased (26) in sciatic nerves compared with those in untreated controls (Figure 6C). A decline in NMNAT2 levels compromises the conversion of NMN into NAD^+^, thus leading to decreased NAD^+^ levels within neuronal axons. Here, we observed a decrease in NAD^+^ levels within sciatic nerves in PAC-treated rats as compared with those in controls (Figure 6D). Importantly, both NMNAT2 and NAD^+^ levels were restored to a control state when treated with 8-AG 72 hours after the last dose of PAC (Figure 6, C and D). A decrease in NAD^+^ can result in an opening of the mitochondrial permeability transition pore (mPTP), as NAD^+^ normally suppresses mPTP opening. We found that PAC results in augmented accumulation of 2’,3’-cAMP in the sciatic nerve, which has been shown to enhance mPTP opening and promote cellular damage (Figure 6E). Treatment with 8-AG normalized these levels.

## Discussion

CIPN is a dose-limiting side effect of chemotherapy drugs that severely impairs function and quality of life, interfering with gait, sleep, daily activities, and mood. Here, we report results on a treatment that may prove effective in reducing PAC-evoked painful neuropathy. Our findings demonstrate that rats exposed to PAC exhibit an increase in proinflammatory/prooxidative purines along with an increase in isoprostanes (an indicator of oxidative stress), axonal degeneration, sensory dysfunction, and alterations in proteins linked with nerve damage and functional decline. Importantly, all PAC-induced changes were blocked and reversed by 8-AG, an endogenous and potent inhibitor of PNPase. Importantly, shRNA knockdown of PNPase significantly reduced PAC-induced sensory dysfunction. Based on the current results, we suggest the concept that PAC-induced CIPN is due to altered purine metabolism, such that inhibition of PNPase prevents or reverses CIPN.

PNPase decreases levels of guanosine and inosine, which are antiinflammatory and neuroprotective purines, while simultaneously generating large amounts of downstream HX and xanthine, which are proinflammatory/prooxidative purines. When HX is metabolized by XO to xanthine and, hence to uric acid, ROS are generated as a byproduct. In fact, XO activation has been shown to promote ROS generation and inflammation, which exacerbates nerve damage and may also be involved in inhibition of neurite outgrowth (27). Not surprisingly, treatments that inhibit oxidation of HX to xanthine suppress inflammatory cytokines and oxidative stress in a number of disorders. Importantly, PAC increases HX and xanthine levels as well as showing a trend toward increased isoprostane levels. Isoprostanes are a group of compounds widely recognized as reliable biomarkers for oxidative stress. These results are consistent with the hypothesis that CIPN is due to a PAC-induced increase in PNPase activity.

The loss of IENFs is a cardinal sign of CIPN and is thought to be responsible for both numbness and pain (3, 28). Our results suggest that IENF loss is due to in part to decreased levels of the enzyme NMNAT2 and the coenzyme NAD^+^. NMNAT2 is a crucial protein necessary for maintaining healthy axons and preventing nerve damage with a deficiency often linked to Wallerian degeneration. Studies show that disruption in NMNAT2 axonal transport can result in spontaneous axonal degeneration (26). In axons, NMNAT2 acts as a protective factor by maintaining adequate NAD^+^ levels (26). PAC can induce activation of the mPTP triggered by mitochondrial calcium overload and high levels of oxidative stress (29). The PAC-induced decline in NAD^+^ (a natural inhibitor of the mPTP) can induce mPTP opening followed by increased ROS generation and mitochondrial dysfunction (29). We have evidence for a PAC-induced increase in 2’,3’-cAMP, which is an intracellular messenger that can intensity this process and open the mPTP (30, 31). High levels of 2’,3’-cAMP (whose production increases with cell injury and stress) can trigger ROS and calcium-dependent mitochondrial swelling. Whether ROS drives changes in these factors that result in a collapse of fibers or contributes to neurodegeneration by indirectly triggering other events such as toxic Ca^2+^ flux requires further investigation.

Though the underlying mechanisms responsible for peripheral nerve damage and loss following PAC treatment are not well understood, increased nitrooxidative stress likely contributes to inflammatory processes that can exacerbate nerve damage and pain (3, 32–34). Furthermore, damaged mitochondria may be a source of ROS that promotes IENF damage and the emergence of spontaneous activity in nociceptive afferents (35). Interestingly, these changes appear to be independent of chemotherapeutic action, as they are associated with a variety of chemotherapeutics with distinct mechanisms of action (36, 37). Support for a “PNPase-based” mechanism of action for 8-AG relies on the ability of 8-AG to restore the balance of a large family of purines, thereby reducing nitrosative oxidative stress and mitochondrial damage, as demonstrated in previous work involving preclinical models of inflammation and aging (38, 39).

While high ROS levels can contribute to nerve damage and hinder regeneration, blocking or scavenging ROS may be beneficial. For example, antioxidant compounds such as α-lipoic acid may improve nerve dysfunction associated with diabetic polyneuropathy (40). In support of a beneficial effect of blocking ROS on nerve regeneration, we showed that 8-AG administration after PAC treatment resulted in significant elevations in IENF expression and expression of GAP-43, a marker of nerve regeneration. In addition, the Ste20-like kinase, Mst3b, plays a key role in axonal regeneration following injury (23). That 8-AG restores the levels of Mst3b is further support for altered purine metabolism as a causal role for IENF loss.

Also consistent with our hypothesis is the observation that Nogo-A is increased in PAC-treated animals. Originally discovered in the context of injury and repair of CNS fiber tracts, Nogo-A is a negative regulator of neurite growth and axonal regeneration. While 8-AG may inhibit Nogo-A expression secondary to the inhibition of PNPase, it is also possible that inhibition may be due to a complex interaction involving Rho-GTPase downstream pathways. That is, there is evidence that 8-AG exhibits a weak ability to inhibit activity of Rac1, a member of the Rho family of small GTPases that plays a complex role in neural damage (41). Inhibition of Rac1 may also ameliorate neuronal oxidative stress damage (42). Nevertheless, the observation that shRNA knockdown of PNPase was associated with comparable results as 8-AG suggests that 8-AG acts through PNPase inhibition.

Patients with CIPN can show a range of predominately sensory symptoms due to the vulnerability of sensory nerve fibers to the toxic effects of anticancer drugs, which can include paresthesia, numbness, and mechanical allodynia (43). Up to 97% of all patients treated with PAC report that their first symptoms including tingling and/or allodynia in fingers and toes as early as 24–72 hours after injection (44). We found that PAC-treated rats exhibited mechanical allodynia as early as 24 hours after injection, and that mechanical allodynia stayed elevated until sacrifice. A study by Boyette-Davis et al. showed that IENF loss coincided with the development of PAC-induced mechanical hyperalgesia (3). Our findings support a causal link between IENF loss and behavior, as 8-AG was not only able to prevent the loss of IENFs as well as corresponding mechanical allodynia, but when given after PAC treatment, was able to restore IENF density and normalize mechanical allodynia to that of control levels. A role of altered PNPase activity in both these changes is supported by our results with pharmacologic inhibition as well as with shRNA knockdown of PNPase.

The enzyme PNPase is likely a pivotal regulator of cellular function such that abnormal expression/activity of PNPase results in a dysregulated purine metabolome that, instead of being protective, becomes deleterious. Our findings suggest that a dysregulation in PNPase that leads to ROS-generating purines leads to a loss of IENF and increased mechanical sensitivity. Accordingly, inhibitors of PNPase would be expected to exert a better therapeutic response compared with inhibitors of ROS production, such as XO inhibitors. This is because PNPase inhibitors activate a “2-hits for 1 drug” mechanism (first hit, higher levels of protective purines; second hit, lower levels of damaging purines). However, the beneficial effects of 8-AG on peripheral nerve structure and function may also involve broader actions through different purine signaling pathways, which help to reduce sources of ROS and modulate immune function and inflammation.

### Conclusions.

Our findings demonstrate that inhibition of PNPase prevents signs of CIPN (increased ROS) and reduces pain behavior. While a large majority of behavioral testing in preclinical CIPN studies utilizes von Frey filaments to assess mechanical allodynia, we recognize that a possible limitation of our study is the use of evoked (reflexive) behavioral testing. As pain is a multidimensional experience, future studies should examine other sensory/discriminative aspects of pain behavior, including temperature (i.e., cold sensitivity), and measures of persistent spontaneous pain, which is an important aspect of CIPN. Whether our findings are unique to PAC treatment or may also apply to other drugs used for treating advanced cancer is not known. Recent studies have proposed the use of PNPase inhibitors for the treatment of cancers, such as breast cancer, making it unlikely that 8-AG would reduce efficacy of PAC and may even enhance its effect on cancer treatment (45). Though 8-AG was effective with regard to protecting and reversing PAC nerve damage, one must be cognizant of potential toxicities associated with this strategy. There is evidence that severe suppression (>90%) of PNPase expression or activity inhibits the proliferation and activation of T cells. However, 8-AG at doses used to treat CIPN provides only partial inhibition of PNPase activity and, therefore, should not suppress T cells. In this regard, preliminary results from our research group (unpublished observations) suggest that treatment of rats with 8-AG has no effect on T cell function even after 6 weeks of treatment, suggesting it is safe to use in patients with cancer, particularly those with compromised immune function. Current drugs and therapies that target the signs and symptoms of CIPN are often ineffective, demonstrating an unmet need for safe and effective treatments for CIPN. Though additional studies are needed to advance 8-AG as a treatment for human CIPN (e.g., demonstrating safety profile and improving life for patients with cancer suffering from nerve damage without hindering cancer treatment), our findings support our hypothesis and point to an approach to reduce the negative effects of PAC-associated CIPN, mitigating sensory dysfunction.

## Methods

### Sex as a biological variable.

Sex was not considered as a variable. Studies were performed in female rats because PAC is widely used for breast and ovarian cancers, and females are more prone to developing CIPN (46).

### Animal model of PAC-induced peripheral neuropathy.

Adult female Harlan Sprague-Dawley rats (3–5 months, 200–225 g, Envigo) were used for all experiments. All rats were given at least 1 week to acclimatize to environmental conditions before any experimental manipulations were conducted. Rats were housed 2 per cage and were given ad libitum access to food and drinking water.

PAC-induced neuropathy was induced by treating rats with PAC (2 mg/kg i.p., prepared from diluting a stock solution of PAC in vehicle [Cremophor EL and ethanol diluted with saline, Teva Pharmaceuticals]) on alternate days (D0, D2, D4, D6) as previously described (34, 47, 48). Control rats received vehicle (Cremophor EL and ethanol diluted with saline) at the same dosing regimen.

### Pharmacologic inhibition of PNPase.

8-AG (5 mg/kg/d, Toronto Research Chemicals) was administered in drinking water, and daily dosing was monitoring; this route of administration was chosen over oral gavage to minimize stress effects associated with the latter route of administration, while also maintaining more consistent systemic levels of the drug. The pharmacologic properties of 8-AG, a potent inhibitor of PNPase, have been well studied (8, 12, 13, 38, 39); it is stable in water and has biochemical and physiological effects when administered via drinking water. This dose of 8-AG was selected based on our preliminary and published studies showing that 5 mg/kg/d provides a urinary concentration of 8-AG of approximately 15 ± 3 μmol/L (mean ± SEM; *n* = 4), a concentration that is at least 5 times the Ki of 8-AG against PNPase (Ki estimated by us to be 2.8 μmol/L against human recombinant PNPase with inosine as substrate) (49). We have tracked the amount of water consumption per day and found no variability (demonstrating consistent dosing). Our published studies demonstrate that 8-AG at 5 mg/kg/d is safe in rats for at least 9 weeks (8). Rats were randomized by cage to groups defined by PAC and 8-AG treatment, such that groups received (a) no PAC and no 8-AG; (b) PAC and no 8-AG; (c) and PAC and 8-AG. 8-AG was started 14 days prior to the start of PAC injections, and animals were euthanized 21 days after the first PAC injection. Importantly, we also examined whether 8-AG can reverse PAC-induced changes with groups that were treated with PAC and then treated with either 8-AG or vehicle 72 hours following the last dose of PAC. All animals were sacrificed on day 21, and tissues used for histological and molecular analysis.

### Molecular inhibition of PNPase.

To achieve in vivo knockdown of PNPase we used lentivirus particles expressing shRNA against rodent PNPase using the Expression Arrest GIPZ lentiviral shRNAmir system developed by Open Biosystems (50). Four days prior to PAC injections, rats were pretreated with lentiviral particles (approximately 10^9^) carrying shRNA directed against PNPase mRNA delivered by injection directly into the sciatic nerve. Sham-treated animals received bilateral injections of viral particles in which the shRNA cassette was replaced with a scrambled sequence. All animals underwent behavioral testing and were sacrificed at day 21, at which point tissues were removed for further analysis. Knockdown was confirmed by Western immunoblotting.

### Behavioral tests for mechanohyperalgesia/allodynia (von Frey).

Mechanical sensitivity was assessed with von Frey filaments applied by the up-down method to the glabrous skin of the rat hind paw (39, 51). Rats were unrestrained in Perspex enclosures positioned over a wire mesh frame (Bioseb) (51, 52). Testing commenced following an acclimatization period of at least 30 minutes, after which exploratory behavior had ended and rats were resting quietly. Each hind paw was tested, starting with the 2 g filament and continuing in ascending order. Once a positive response was observed (flinch or lift), the preceding lower force filament was tested, and again the experimenter continued to increase the force of the filament until another positive response was observed and/or at least 4 filaments were tested following the initial response. From these patterns, the 50% paw withdrawal threshold (the force that elicits a withdrawal response 50% of the time) was calculated using a previously developed formula (51, 52), whereby the 50% threshold (g) = 10^(X^^+^^kd)^/10^4^, where *X* equals the value (in log units) of the final von frey filament, *k* equals tabular value for the response pattern, and *d* equals the average increment (in log units) between von Frey filaments. The advantage of this approach is that it lends itself to the use of parametric statistics for analysis. Testing was performed by a single experimenter, and the investigator collecting data was blinded to treatment group assignment.

### Measurement of purines and NAD^+^.

Lyophilized nerve tissue becomes spongy and, hence, can be quickly saturated by organic solvents in extraction medium. Therefore, nerve tissue samples were lyophilized, and the tissue dry weight was estimated. The lyophilized tissue was dispersed with a Polytron homogenizer in ice-cold extraction medium composed of methanol, water, and acetonitrile (2:2:1) and containing heavy-labeled internal standards. Next, the tissue homogenate was extracted by chloroform under nitrogen to remove lipids and to release tightly bound metabolites. The lipid-free extract of water-soluble metabolites was additionally purified using HLB solid-phase extraction cartridges (Waters). Analytes were measured using UPLC-MS/MS, as we previously described (39, 53), with the exceptions that the mobile phase was water/acetonitrile instead of water/methanol and the column was an ACQUITY UPLC Premier HSS T3 Column VanGuard fit, 100 A, 1.8 m, 2.2 mm (Waters) instead of a BEH C18 column. Data were quantified as ng of metabolite normalized by mg tissue dry weight.

### Measurement of isoprostanes.

The 8-epimer of prostaglandin F2α (8-iso-PGF2α) is an isoprostane produced by the peroxidation of arachidonic acid in membrane phospholipids and is a validated biomarker of oxidative stress (54). Isoprostane levels in sciatic nerve tissue were measured by a competitive immunoassay (Enzo Life Sciences, ADI-900-091) following the manufacturer’s instructions. Protein extracts were subjected to hydrolysis with 1-part 10 N NaOH for 4 parts sample at 45°C for 2 hours for the measurement of both free and esterified 8-iso-PGF2α. Following neutralization with HCl, a 75 μg sample was incubated with polyclonal anti-8-iso-PGF2α and alkaline phosphatase conjugated 8-isoPGF2α. After an overnight incubation at 4°C, the excess reagents were washed from the assay, and substrate was added. After a short incubation time, the enzyme reaction was stopped and absorbance at 405 nm was measured by spectrophotometry using a Tecan Spark microplate reader. Data were quantified as pg of 8-iso-PGF2α normalized by mg protein.

### Immunofluorescence.

The pan-neuronal marker, PGP9.5 was used to assess changes in IENF density (3, 55). Animals were sacrificed after behavioral testing on day 21, and a section of glabrous skin was excised from the wide part of the plantar hind paw that lies distal to the calcaneus and proximal to the digital tori (47). Glabrous skin biopsies were immediately placed overnight in fixative (containing 0.25% paraformaldehyde, 94 mM lysine, and 10 mM sodium periodate in 0.1 M phosphate buffer, pH 7.2), cryoprotected (30% sucrose-phosphate buffered saline), and embedded and frozen in Tissue-Tec Optimal Cutting Temperature Compound (OCT, Sakura Finetech). To assess IENF density, 30 μm serial sections were cut on a cryostat (Leica) perpendicular to the epidermis and mounted on gelatin-coated slides (Fd Neurotechnologies Inc.). Nonspecific staining was blocked using 10% normal donkey serum (Sigma-Aldrich) in PBS containing 0.5% triton X-100 (Sigma-Aldrich) at room temperature for an hour. Sections were then incubated overnight at 4°C using antibodies (validated in rat tissue) against neuron specific ubiquitin hydrolase, PGP9.5 (neuron-specific protein; Invitrogen, PA52012, 1:500) or GAP-43 (labels IENF regenerating nerve sprouts, associated with ongoing pain; Millipore Chemicon, AB5220, 1:1,000) (56) in the same blocking buffer. Nonspecific immunostaining was assessed in sections incubated overnight in blocking buffer without primary antibodies. Following washes with PBS, the slides were incubated with donkey anti-rabbit Alexa Fluor 488 (Invitrogen, A32790) diluted 1:1,000 in blocking buffer for 2 hours at room temperature. After washing with PBS, nuclei were counterstained with DAPI (Invitrogen D1306, 1:5,000), coverslipped with ProLong Diamond antifade mountant (Invitrogen, P36961), and imaged using an Olympus BX63 microscope using CellSense software (Olympus). IENF analysis was calculated from PGP9.5 immunofluorescence as follows. Branching nerve fibers which split within the epidermis were counted as one, while branching nerve fibers which split within the dermis were counted individually. (57–59). Values were normalized to the longitudinal length of epidermis (μm) in each field. For IENF quantification, we selected a minimum of 5 plantar skin sections per animal with 3 sites for each section. This method reliably demonstrates a dermal plexus of nerve fibers parallel to the skin considered to be unmyelinated axons carrying pain sensation. GAP-43 immunofluorescence was quantified as follows. Sections to be compared were prepared and imaged concurrently using identical acquisition settings, and images were exported into ImageJ (NIH) software for analysis. Images were converted into 8-bit, and the DAPI and GAP-43 fluorescence channels were split. Mean fluorescence in a background area was measured and subtracted from each channel. Thresholding was standardized using IJ IsoData and Renyi Entropy auto-threshold functions for GAP-43 and DAPI fluorescence, respectively. The area of the dermis and epidermal junction was selected in each image, and the mean fluorescent intensity within the area was measured. For each image, the mean GAP-43 fluorescence was normalized to the mean DAPI fluorescence of the same area. For GAP-43 quantification, we selected a minimum of 5 plantar skin sections per animal with 3 sites for each section.

### Transmission electron microscopy.

After sacrifice, sciatic nerves were removed and fixed in room temp 2.5% glutaraldehyde (25% glutaraldehyde stock EM grade, Polysciences) in 1x PBS (10x Phosphate Buffered Saline, Fisher), pH 7.3. The specimens were rinsed in PBS, post-fixed in 1% osmium tetroxide (Osmium Tetroxide crystals, Electron Microscopy Sciences) with 1% potassium ferricyanide (Potassium Ferricyanide), dehydrated through a graded series of ethanol (30%–90% Reagent Alcohol, Fisher and 100% Ethanol 200 Proof, Pharmco), and propylene oxide (propylene oxide, Electron Microscopy Sciences) with a graded series of infiltration steps, and embedded in Poly/Bed 812 (Glauert formulations) (Dodecenyl Succinic Anhydride, Sigma-Aldrich and Nadic Methyl Anhydride, Poly/Bed 812 Resin and BDMA [Benzyl dimethyl amine], Polysciences) under vacuum. Semithin (300 nm) sections were cut on a Leica Reichart Ultracut (Leica Microsystems), stained with 0.5% Toluidine Blue in 1% sodium borate (Toluidine Blue O and Sodium Borate, Fisher), and examined under the light microscope. Ultrathin sections (65 nm) were stained with 2% uranyl acetate (Uranyl Acetate dihydrate, Electron Microscopy Sciences) and Reynold’s lead citrate (Lead Nitrate, Sodium Citrate, and Sodium Hydroxide, Fisher) and examined on a JEOL 1400 Flash transmission electron microscope with AMT Biosprint 12, 12mp camera (Advanced Microscopy Techniques).

### Western immunoblotting.

Sciatic nerves (taken from distal trifurcation to the origination of the pudendal nerve) were homogenized using Lysing Matrix D in a FastPrep 24 instrument (MP Biomedicals) in HBSS (5 mM KCl, 0.3 mM KH_2_PO_4_, 138 mM NaCl, 4 mM NaHCO_3_, 0.3 mM Na_2_HPO_4_, 5.6 mM glucose, and 10 mM HEPES, pH 7.4) containing complete protease inhibitor cocktail (1 tablet/10 mL, Roche) and phosphatase inhibitor cocktail (Sigma-Aldrich, 1:100). After centrifugation (16,200*g*; 15 min at 4ºC), the membrane protein fraction was prepared by suspending the membrane pellets in lysis buffer containing 0.3 M NaCl, 50 mM Tris-HCl (pH 7.6) and 0.5% Triton X-100 and the same concentration of protease inhibitors as above. The suspensions were incubated on ice and centrifuged (16,200*g*; 15 min at 4ºC). The protein concentrations of the combined supernatants were determined using the Pierce BCA protein assay (Thermo Scientific). After denaturation (100ºC for 5 min) in the presence of Laemmli sample buffer, lysate from each sample was separated on a 4%–15% TGX Stain-Free SDS-PAGE gel (Bio-Rad). As a reliable loading control, total protein measurement per sample was determined using Bio-Rad Stain Free SDS-PAGE gel technology. UV-activated protein fluorescence was imaged on a ChemiDoc MP (Bio-Rad). After proteins were transferred to polyvinylidene fluoride membranes, the membranes were incubated in 5% (w/v) dried milk dissolved in TBS-T (20 mM Trizma, 137 mM NaCl, 0.1% Tween-20, pH 7.6), rinsed with TBS-T, and incubated overnight at 4ºC with primary antibody, including GAP-43 (Millipore Chemicon, AB5220, 1:1,000), NOGO-A (Cell Signaling Technology, 13401, 1:2,000), Mst3b (Cell Signaling Technology, 4062S, 1:1,000), NMNAT2 (Santa Cruz Biotechnology, SC-515206, 1:500), and PNPase (Atlas Antibodies, HPA001625, 1:2,000). After washing in TBS-T, the membranes were incubated with the appropriate secondary antibody (sheep anti-mouse HRP, Southern Biotech, or donkey anti-rabbit HRP, Advansta) for 1 hour in 5% (w/v) milk TBS-T, washed, incubated in WesternBright Quantum (Advansta), and then imaged on a ChemiDoc MP (Bio-Rad). The volume (intensity) of each protein species was determined and normalized to total protein using Image Lab software (Bio-Rad).

### Statistics.

Data were analyzed in GraphPad Prism 10 by a 1-way or 2-way ANOVA followed by a Fisher’s least significant difference test between groups. *P* < 0.05 was considered significant. Data are shown as mean ± SD. Data are available in the Harvard Database Repository, which uses the Dataverse Project open-source research data repository software (https://doi:10.7910/DVN/EJAPIM), in the paper’s supplemental material (available online with this article; https://doi.org/10.1172/jci.insight.202639DS1), or from the corresponding author upon request.

### Study approval.

The Institutional Animal Care and Use Committee of the University of Pittsburgh approved all procedures. The investigation conformed to the guidelines of the *Guide for the Care and Use of Laboratory Animals* published by the US NIH (National Academies Press, 2011).

### Data availability.

Data are be available in the Harvard Database Repository, which uses DataverseProject open-source research data repository software (https://doi.org/10.7910/DVN/EJAPIM), Harvard Dataverse, V1, in the paper’s supplemental material, or from the corresponding author upon request.

## Author contributions

Designed research studies: LAB, EKJ, SCW, and AJK. Conducted experiments: AWJ, VBR, MLGS, and JF. Acquired data: AWJ, VBR, MLGS, and JF. Provided reagents and developed methodologies: LAB, EKJ, SCW, and AJK. Wrote manuscript: LAB and EKJ. Interpreted data: LAB, EKJ, SCW, AJK, and AWJ.

## Funding support

This work is the result of NIH funding, in whole or in part, and is subject to the NIH Public Access Policy. Through acceptance of this federal funding, the NIH has been given a right to make the work publicly available in PubMed Central.

- NIH R01 DK135076 (to LAB and EKJ).
- NIH U54 DK137329 (to EKJ).
- Richard King Mellon Foundation Award (to LAB).

## Supplementary Material

Unedited blot and gel images

Supporting data values

## Version 1 04/08/2026

Electronic publication
