# Supplementary material for: 8-Aminoguanine protects against paclitaxel-induced neural degeneration and mechanical allodynia
Source: JCI Insight. 2026 Apr 8;11(7):e202639. doi: 10.1172/jci.insight.202639 (PMC13134735; doi:10.1172/jci.insight.202639)
Supplement: Unedited blot and gel images [file jciinsight-11-202639-s254.pdf]

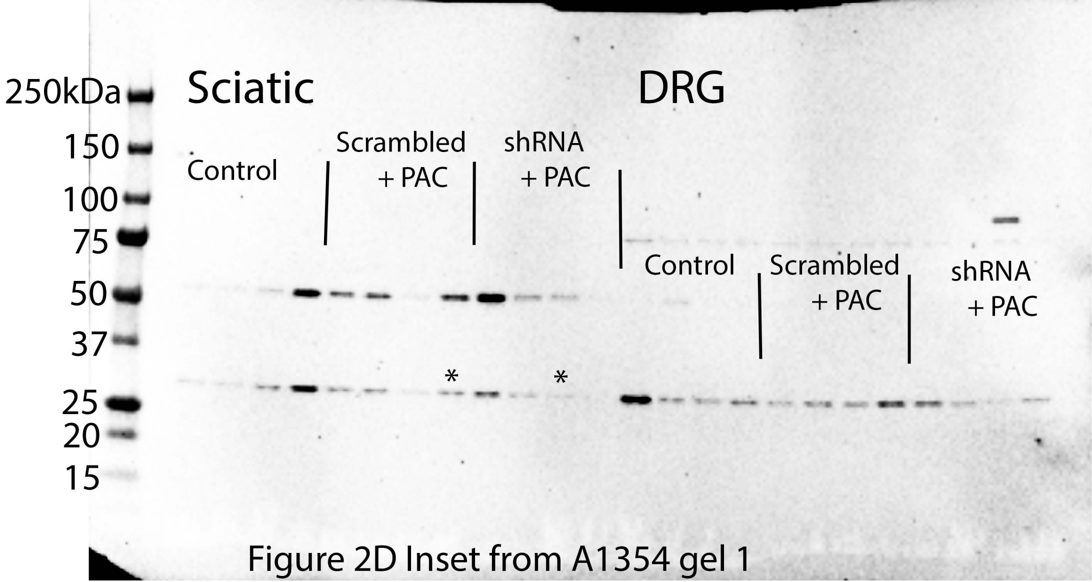

Figure 2D Inset from A1354 gel 1
PNPase, Atlas Antibodies HPA 001625
Rabbit polyclonal at 1:1000 32kDa

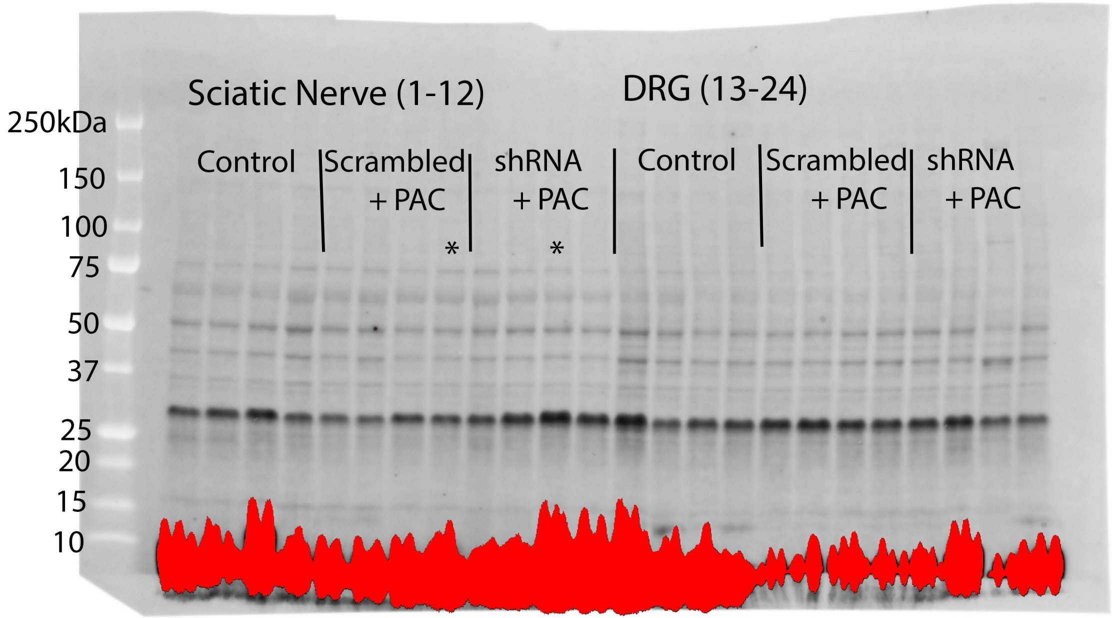

Figure 2D inset
A1354 gel 1 stain free blot

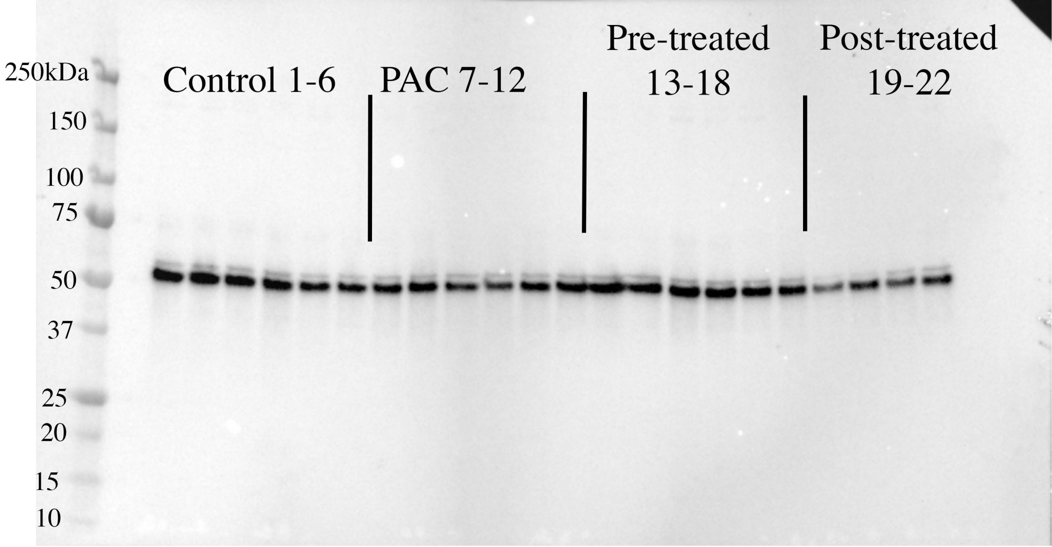

Figure 5E: A1316 gel 3 GAP43 (50kDa) in sciatic nerve
Millipore Chemicon AB5220 Rabbit polyclonal at 1:5000

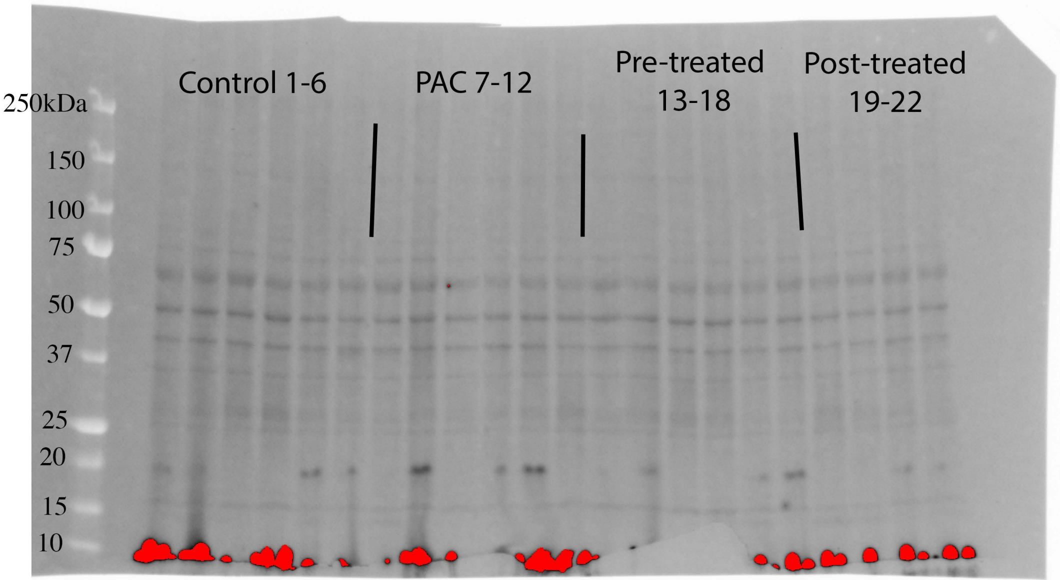

Figure 5E: A1316 stain free blot for GAP43

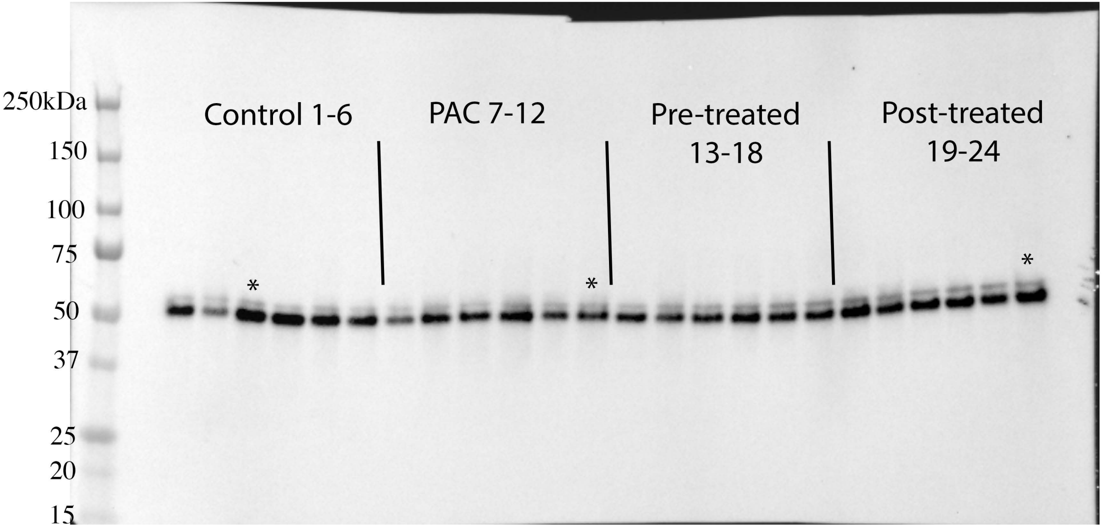

Figure 5E: A1319 gel 1 GAP43 (50kDa) in sciatic nerve
Millipore Chemicon AB5220 Rabbit polyclonal at 1:5000

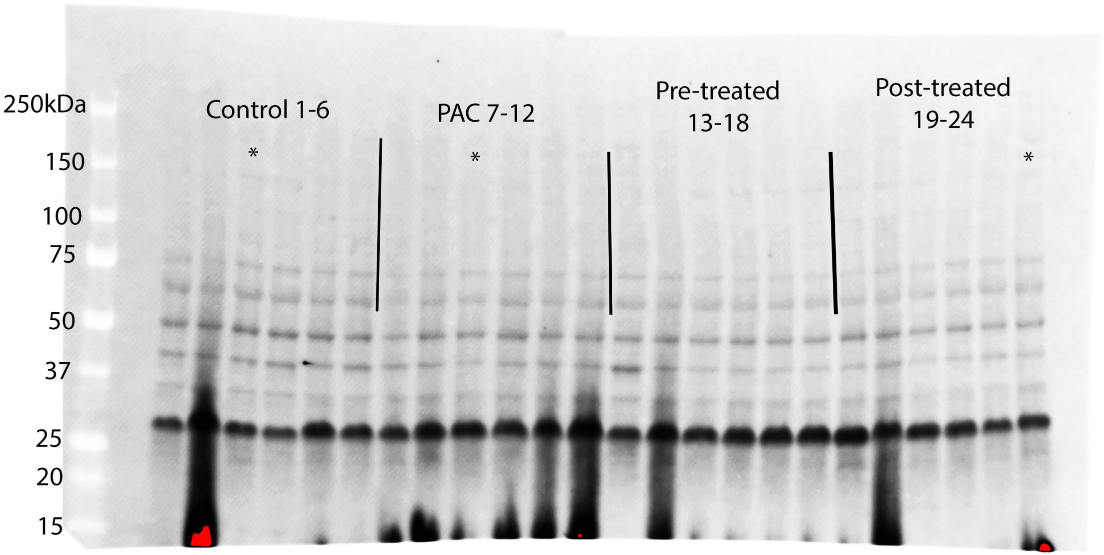

Figure 5E: A1319 stain free blot for GAP43

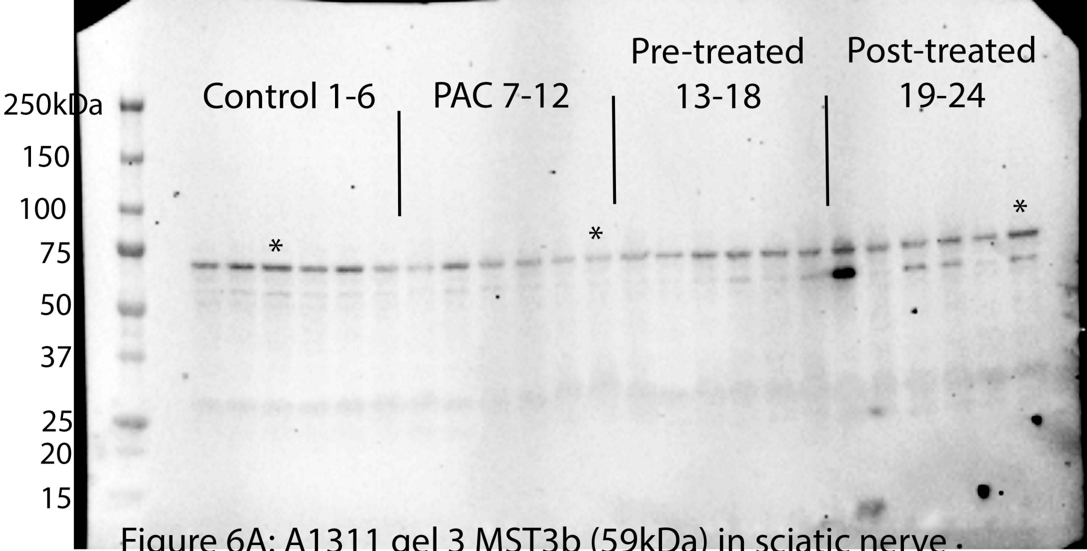

Figure 6A: A1311 gel 3 MST3b (59kDa) in sciatic nerve
Cell Signaling 4062S Rabbit polyclonal 1:1000

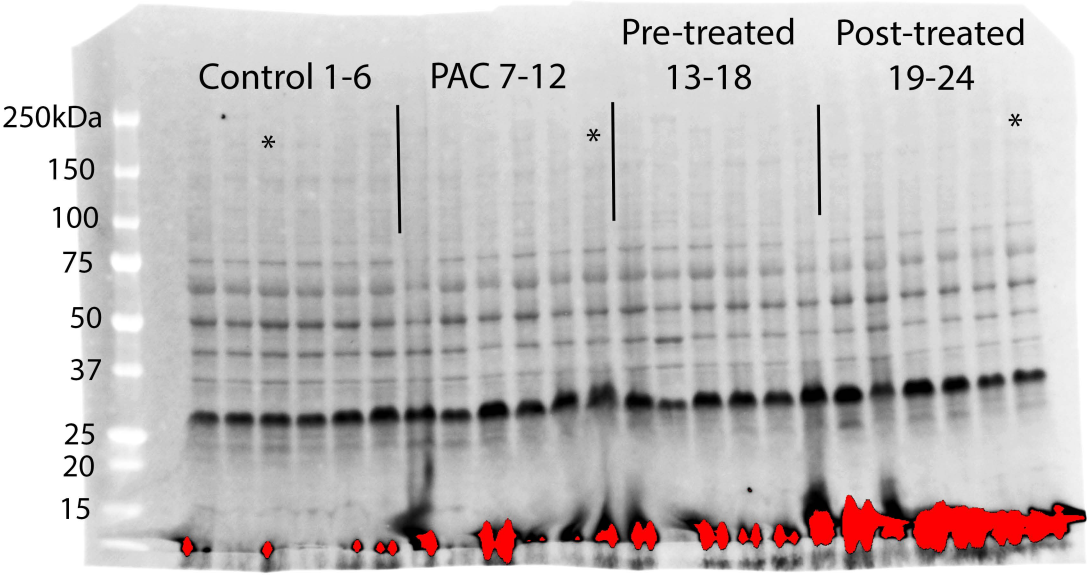

Figure 6A: A1311 gel 3 stain free blot for MST3b

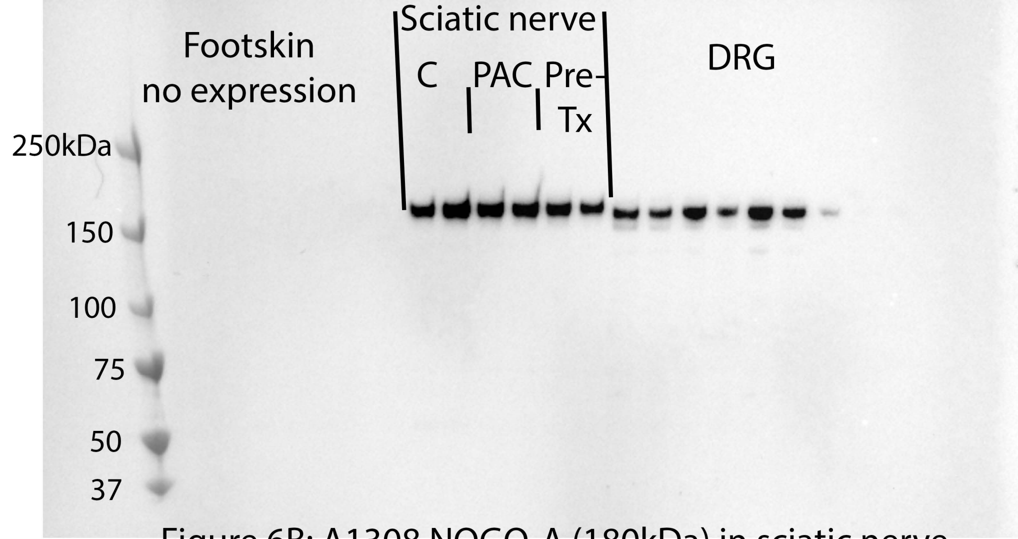

Figure 6B: A1308 NOGO-A (180kDa) in sciatic nerve
Cell Signaling 13401 Rabbit polyclonal at 1:2000

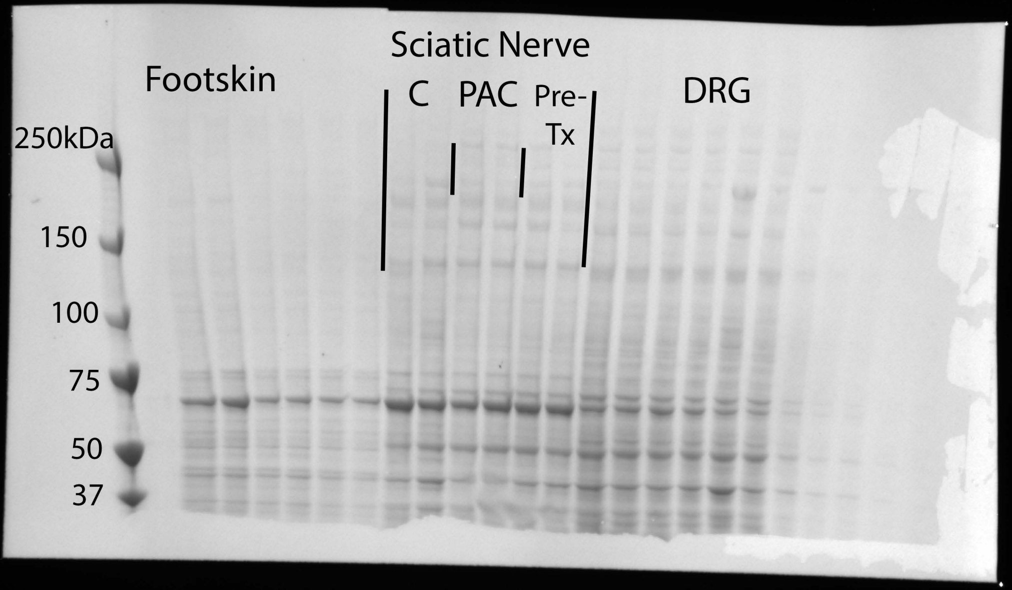

Figure 6B: A1308 protein stain for NOGO-A

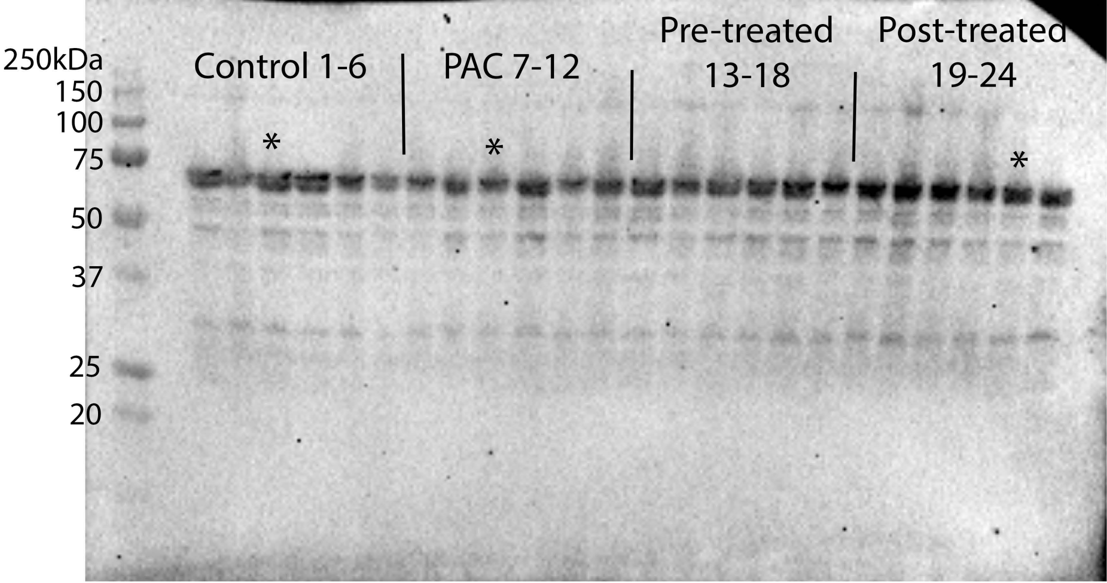

Figure 6C: A1321 gel 3 NMNAT2 (34kDa, 68kDa dimer) in sciatic nerve
Santa Cruz SC-515206 Mouse monoclonal at 1:500

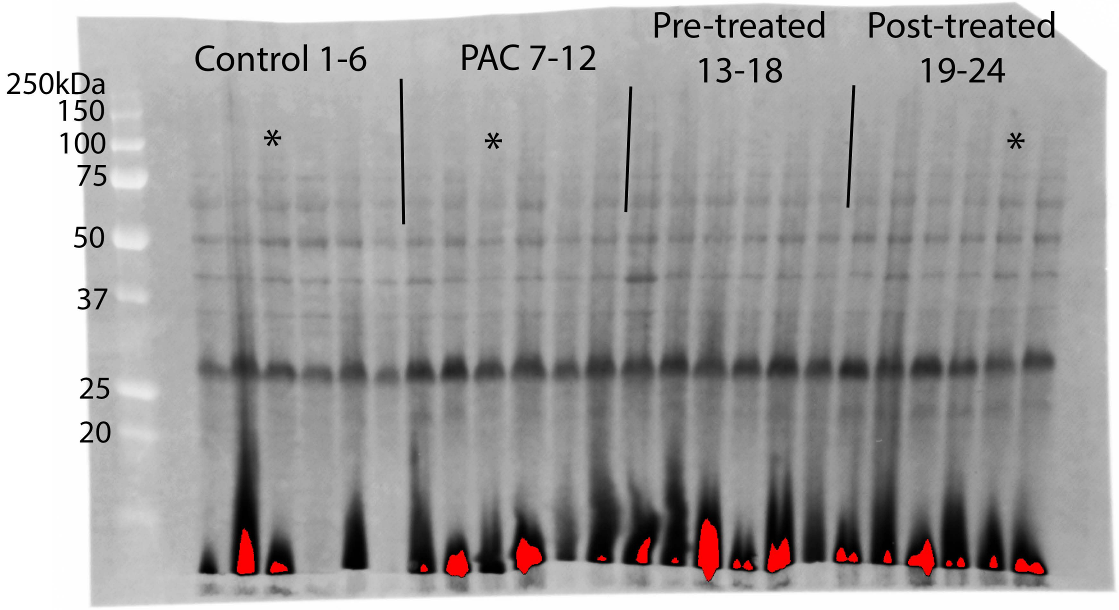

Figure 6C: A1321 gel 3 stain free blot for NMNAT2
